# Mechanical Thrombectomy in Isolated Occlusion of the Proximal Posterior Cerebral Artery

**DOI:** 10.3389/fneur.2021.697348

**Published:** 2021-07-29

**Authors:** Christian Herweh, Mohamad Abdalkader, Thanh N. Nguyen, Volker Puetz, Daniela Schöne, Daniel Kaiser, Chih-Hao Chen, Jiann-Shing Jeng, Markus A. Möhlenbruch, Peter A. Ringleb, Simon Nagel

**Affiliations:** ^1^Department of Neuroradiology, Heidelberg University Hospital, Heidelberg, Germany; ^2^Division of Interventional Neurology/Neuroradiology, Boston Medical Center, Boston, MA, United States; ^3^Department of Neurology, Dresden University Hospital, Dresden, Germany; ^4^Institute of Neuroradiology, University Hospital Carl Gustav Carus, Dresden, Germany; ^5^Department of Neurology, National Taiwan University Hospital, Taipei, Taiwan; ^6^Neurology, Heidelberg University Hospital, Heidelberg, Germany

**Keywords:** posterior circulation stroke, posterior cerebral artery, endovascular therapy, mechanical thrombectomy, best medical management

## Abstract

**Introduction:** Endovascular therapy (EVT) is established as first-line treatment for acute ischemic stroke (AIS) due to large vessel occlusion (LVO) in the anterior circulation. For basilar artery occlusion, recent randomized clinical trials demonstrated not only equipoise but also advantages for EVT under particular circumstances. It remains unclear whether EVT offers an advantage over best medical management (BMM) including thrombolysis (IVT) in isolated occlusion of the proximal posterior cerebral artery (PCAO).

**Methods:** Patients with AIS due to PCAO proven by CT or MR angiography were retrospectively identified from local databases at four comprehensive stroke centers in Germany, USA, and Taiwan between 2012 and 2020. Demographic and clinical data were collected, and imaging characteristics including pretherapeutic, interventional, and follow-up imaging were reviewed locally at each center. Patients were grouped according to therapy, i.e., BMM including IVT alone vs. BMM and EVT. Efficacy endpoints were early neurological improvement (ENI) after 24 h or at discharge, good outcome (modified Rankin scale 0–2) after 3 months, as well as hemorrhagic complications and in-house deaths as safety endpoints.

**Results:** We included 130 patients of whom 23 (17.7%) received EVT. EVT patients had more proximal occlusions (69.9 vs. 43%, *p* = 0.023) and had a better premorbid function [premorbid mRS, 0 (0–4) vs. 1 (0–3), *p* < 0.01] when compared to BMM patients. IVT showed a trend toward being less performed in the EVT group (21.7 vs. 41.1%, *p* = 0.1), while other baseline parameters were balanced. Successful reperfusion was achieved in 52% of EVT patients. ENI was more frequent in the EVT group (61 vs. 35.5%, *p* = 0.034). Good outcome at 90 days and safety endpoints did not differ. In a bivariate analysis, ENI was independently predicted by the use of EVT (OR, 2.76; CI, 1.055–7.04) and the baseline National Institutes of Health Stroke Scale (NIHSS) (OR, 1.082; CI, 1.027–1.141 per point increase).

**Discussion:** EVT in isolated PCAO appears safe and feasible. Positive effects on clinical outcome are primarily on ENI but also depend on the initial stroke severity. Further prospective or randomized studies are needed to better describe the potential long-term clinical benefits of EVT for PCAO as compared with best medical management.

## Introduction

Endovascular therapy (EVT) is a standard of care for patients with acute ischemic stroke (AIS) due to large vessel occlusions (LVO) in the anterior circulation. Although EVT was pioneered in basilar artery (BA) occlusion (1), evidence for the benefit of EVT in BA occlusion (BAO) could not be concluded, yet. Recent randomized clinical trials (RCTs) failed to meet the primary endpoint of good outcome after 90 days but demonstrated benefit in a subgroup of severely affected patients or in the as-treated population (2, 3). There is a profound difference between the anterior and posterior circulation in functional vascular anatomy, topography, and pathophysiology of stroke. Ischemic damage in vascular territories of proximal perforating arteries in the posterior circulation, i.e., brainstem and diencephalon, has a greater influence on neurological function and the National Institutes of Health Stroke Scale (NIHSS) compared to infarction in the rostral basal ganglia, i.e., caudate nucleus and putamen. Here, infarction occurs regularly even after clinically successful EVT (4, 5) because these areas are particularly vulnerable due to reduced collateral blood supply (6). At the same time, cortical areas supplied by the anterior circulation, e.g., the central region, impact the NIHSS much more than those supplied by the posterior circulation, i.e., in the occipital lobe. This topology also applies to the posterior cerebral artery. Here, perforating arteries originating proximally provide supply to the cerebral peduncle and dorsolateral thalamus. Nevertheless, motor deficits are reported only in about 30% of acute, isolated posterior cerebral artery (PCA) occlusion (7–9).

Therefore, we investigated whether EVT can have beneficial effects on immediate and long-term functional outcome in patients with AIS caused by primary, isolated occlusion of the PCA in comparison to best medical management (BMM), with or without concomitant thrombolysis.

## Patients and Methods

In four centers, two in Germany, one in the USA, and one in Taiwan, respectively, patients consecutively treated between 2012 and 2020 for acute ischemic stroke (AIS) with isolated occlusion of the posterior cerebral artery were retrospectively identified from prospectively maintained stroke databases. Inclusion criteria were (1) occlusion of the proximal posterior cerebral artery in the P1 segment, at the P1/P2 junction or in the P2 segment proven by either CT angiography (CTA) or magnetic resonance angiography (MRA), and (2) causing acute focal neurological symptoms (NIHSS >0). Exclusion criteria were (1) severe preexisting disability defined as modified Rankin scale (mRS) >4 prior to index stroke; (2) secondary PCA occlusion after reperfusion of BAO, spontaneously or after therapy; (3) bilateral occlusion of the PCA or its branches; (4) peripheral PCA occlusions, i.e., beyond the P2 segment; and (5) concomitant occlusion in the anterior circulation, i.e., internal carotid artery (ICA), middle cerebral artery (MCA), or anterior cerebral artery (ACA) or their branches. Imaging protocols [computed tomography (CT) or magnetic resonance imaging (MRI) and perfusion imaging] and treatment decision followed local standard operation procedures (SOPs) at each center. Demographic data, comorbidities (including diabetes mellitus, previous stroke, smoking, and atrial fibrillation), previous anticoagulation or antiplatelet therapy, initial National Institute of Health Stroke Scale (NIHSS), prestroke mRS, time intervals to stroke therapy, intravenous thrombolysis therapy (IVT) using recombinant tissue plasminogen activator (rtPA), location of vessel occlusion, and presence of intracerebral hemorrhage (ICH) defined according to the Heidelberg Bleeding Classification (HBC) (10) were collected. ICH was rated as symptomatic if there was a clinical deterioration resulting in an NIHSS increase, which was accountable to the bleeding. All clinical scores were obtained by NIHSS/mRS-certified neurologists. The clinical outcome as per mRS 90 days after stroke onset was obtained by a standardized interview (unblinded investigator per phone call or a personal letter to the patient) or patient assessment during follow-up in clinic. Reperfusion was described according to the modified thrombolysis in cerebral infarction (mTICI) scale and grouped in a simplified scheme, i.e., complete (3 and 2b) or no reperfusion (0–2a) in the territory of the posterior cerebral artery (11). Mismatch on perfusion imaging between time to peak (TTP) and cerebral blood volume (CBV) maps or diffusion weighted imaging (DWI) was only semiquantitatively assessed and defined as described in the European Cooperative Acute Stroke Study (ECASS) IV trial protocol with a TTP to infarct core (DWI, CBV) ratio of 1.2 (>20 mL) (12).

The primary efficacy endpoint was early neurological improvement (ENI) defined as a decrease in four or more points compared to baseline NIHSS or zero points on the earliest NIHSS score after 24 h but before discharge in accordance with a recently published study (13). The secondary endpoint was good outcome, defined as mRS 0–2 after 90 days. Safety endpoints were in-house deaths, fatal ICH, and asymptomatic ICH. Ethical approval was obtained at the local centers' institutional review boards that contributed the patients to the pooled analysis with anonymized data.

### Statistical Analysis

The statistical analysis followed an exploratory design, and the patient cohort was described using summary measures of the empirical distribution. Continuous variables are given as means (SD) and/or median [interquartile range (IQR) and range, as appropriate]. Nominal variables are described as absolute and relative frequencies. Patients were first grouped according to the mode of therapy, i.e., BMM or BMM incl. EVT, and parameters were compared between groups. In general, continuous variables were tested using a *t*-test for independent groups, and ordinal variables were compared with the Mann–Whitney test, whereas categorical variables were compared using the chi^2^ test. Since this was a retrospective data analysis, all *p*-values are to be interpreted in a descriptive sense; *p* < 0.05 were denoted as significant. To identify factors that were associated with the primary outcome endpoint, patients were also compared according to ENI. After univariate analysis, selected variables with *p* ≤ 0.1 were included in a logistic regression model with a stepwise forward approach for independent predictors of ENI. All statistical analyses were performed using SPSS27®.

Anonymized data are available by reasonable request to the corresponding author.

## Results

We included 130 patients with a median age of 77.5 years (IQR, 66–82) who presented 137 (median; IQR, 73.5–268.5) min after symptom onset, which was unwitnessed in 38 (29.2%) of patients. Sixty patients (46.2%) were female, 62 (47.7%) had proximal occlusions (i.e., in the P1 segment), while the remaining occlusions were distal to P1. All PCAs originated from the basilar tip. Most patients (117/90%) were admitted directly and had a median NIHSS of 7 (IQR, 3–12). Premorbid disability (mRS > 2) was present in 22 (16.9%) of patients, and the median premorbid mRS was 1 (range, 0–4). There was a weak but significant correlation between the premorbid mRS and the NIHSS on admission (*r* = 0.2; *p* = 0.02; Spearman rank). Imaging was primarily performed with computed tomography (CT) in 118 (91%) and CT angiography in 106 (81.5%) of the patients, while 32 (24.6%) had an MRI (21 patients had both consecutively, i.e., CT and MRI). Perfusion imaging with either MRI or CT was performed in 31 (23.8%) of the patients, and 23 (74%) of them had a visual mismatch, either on MRI (6) or on CT (14).

Twenty-three patients received EVT (17.7%) with a mean door-to-groin time of 122 ± 100 min of whom five patients also received IVT. Overall, IVT was performed in 49 (37.7%) of the patients with a mean door-to-needle time of 49 (25–78.5) min. BMM without any acute reperfusion therapy was utilized in 63 (48.5%) of patients.

In the BMM group, significantly more patients had typical vascular risk factors, i.e., hypertension, diabetes, and hypercholesterolemia (see Table 1). The prestroke Rankin scale was significantly higher in the conservative group. Patients in the EVT group had more proximal occlusions, had less CT imaging, and had more perfusion imaging, irrespective of the imaging modality. Although there was a non-significant trend for EVT patients to arrive later in the hospital, subsequently, they received IVT faster than those who did not undergo EVT. Other parameters were balanced between both groups. Of 23 patients who received EVT, 13 (54%) experienced complete or near-complete reperfusion (TICI 2b−3) with 2 (16%) of them exhibiting a residual stenosis of the target vessel of more than 50%, while five patients had only partial recanalization with the majority (4/5) harboring a residual stenosis (see Table 2). In the remaining six patients, no successful recanalization could be achieved.

**Table 1:** Baseline clinical and imaging characteristics.

**Baseline clinical data**	**BMM + EVT**	**BMM**	***p*-value**
	***n* = 23**	***n* = 107**	
Sex (female)	9 (39.1%)	51 (47.7%)	0.49^χ^
Age, mean ± SD	70 ± 13.3	74 ± 13.1	0.19^t−*test*^
Mothership (vs. “drip & ship”)	21 (91.3%)	96 (89.7%)	1.0^χ^
Wake-up stroke	7 (30.7%)	31 (29%)	1.0^χ^
Premorbid mRS, median (range)	0 (0–4)	1 (0–3)	<0.01^MWU^
Premorbid disability (mRS > 2)	1 (4.3%)	21 (19.6%)	0.12^χ^
NIHSS on admission, median (range)	9 (1–20)	7 (1–38)	0.15^MWU^
Hypertension	16 (69.6%)	97 (90.7%)	0.013^χ^
Diabetes	4 (17.4%)	48 (44.9%)	0.018^χ^
Hypercholesterolemia	8 (36.4%)	72 (67.3%)	<0.01^χ^
Current smoking	5 (20%)	17 (15.9%)	0.54^χ^
Previous stroke	6 (26.1%)	29 (27.1%)	1.0^χ^
Coronary heart disease	8 (34.8%)	26 (24.3%)	0.31^χ^
Atrial fibrillation	6 (26.1%)	41 (38.7%)	0.34^χ^
Oral anticoagulation	4 (17.5%)	24 (22.6%)	0.78^χ^
Mean arterial pressure	110 ± 22	112 ± 17	0.74^t−*test*^
**Baseline radiological findings**			
Occlusion location, proximal	16 (69.9%)	46 (43%)	0.023^χ^
CT performed	18 (78.3%)	100 (93.5%)	0.038^χ^
MRI performed	5 (21.7%)	27 (25.2%)	1.0^χ^
Perfusion imaging performed (CTP or MRP)	10 (43.5%)	21 (19.6%)	0.03^χ^
MRP/CTP mismatch > 20%	9/10 (90%)	14/21 (70%)	0.37^χ^

χ *, chi-square test; MWU, Mann–Whitney test*.

**Table 2:** Procedural and outcome parameters.

**Procedural parameters**	**BMM + EVT (23)**	**BMM (107)**	***p*-value**
intravenous thrombolysis	5 (21.7%)	44 (41.1%)	0.1^χ^
Time-to-treatment (time from symptoms onset to IVT or to Groin Puncture) in min, mean ± SD	353 ± 263	246 ± 210	0.13^t−*test*^
Onset to door (EVT center) in min, mean ± SD	281 ± 223	159 ± 115	0.061^t−*test*^
Door to needle time in min, mean ± SD	20 ± 13	61 ± 35	<0.01^t−*test*^
Door to groin time in min, mean ± SD	122 ± 100	n.a.	n.a.
**Outcome parameters**			
Recanalization complete or near complete (TICI 2b-3)	13/23 (56%)	n.a.	n.a.
Residual artery stenosis	4/23 (17%)	n.a.	n.a.
NIHSS drop, median (IQR)	4 (−1–7)	2 (0–4)	0.29^MWU^
ENI	14 (61%)	38 (35.5%)	0.034^χ^
mRS at day 90, median (range)	3 (0–6)	3 (0–6)	0.57^MWU^
Good outcome	10 (43.5%)	45 (42.1%)	1.0^χ^
Good outcome incl. return to baseline mRS	10 (43.5%)	55 (51.4%)	0.65^χ^
Excellent outcome (mRS 0–1 at 90 days)	4 (17.4%)	23 (21.5%)	0.78^χ^
Symptomatic ICH	1 (4%)	3 (3%)	0.55^χ^
Fatal ICH	0%	1 (0.9%)	1.0^χ^
In-house deaths	1 (4.3%)	5 (4.7%)	1.0^χ^
Death at day 90	3 (13%)	8 (7.5%)	0.41^χ^

*BMM, best medical management; EVT, endovascular therapy; ENI, early neurological improvement; ICH, intracranial hemorrhage*;

χ *, chi-square test, MWU, Mann–Whitney test*.

The primary endpoint ENI was significantly more frequent in EVT patients. The NIHSS dropped in median by four points (IQR, 1–7) in the EVT group and by two points (IQR, 0–4) in the BMM group (*p* = 0.29). Long-term outcome parameters, however, did not differ; the median mRS at 90 days was 3 (0–6; *p* = 0.57) in both groups, and the proportion of patients with good outcome (mRS 0–2) was 43.5% in the EVT and 42.1% (*p* = 1.0) in the BMM group, which increased to 51.4% in the BMM group but was unchanged in the EVT group when patients who returned to their prestroke mRS level (*p* = 0.65) were included (see Table 2). Moreover, more patients with ENI tended to have a good outcome. In the EVT group, 6 (26%) patients had any ICH, while this occurred in 14 (13%) in the conservative group (*p* = 0.12). There was one mildly symptomatic parenchymal hemorrhage i.e., resulting in a drop of two points on the NIHSS in the EVT group. In the conservative group, there were two cases of symptomatic intracranial hemorrhage, one parenchymal and one subarachnoid resulting in an NIHSS increase of 9 and 6 points, respectively, and one case of fatal ICH. All patients had received thrombolysis. There were no angiographic signs of vessel perforation and one asymptomatic subarachnoid bleeding on follow-up CT in the EVT group but none in the conservative group (for details, refer to Table 3). There was no difference between therapy groups in fatality rates neither for in-house deaths (4.3 vs. 4.7%; *p* = 1.0) nor after 90 days (13 vs. 7.5%; *p* = 0.41).

**Table 3:** ICH distribution according to the Heidelberg Bleeding Classification.

		**BMM + EVT (*n* = 23)**	**BMM (*n* = 107)**
Heidelberg Bleeding Classification	1a	1	5
	1b	2	5
	1c	1	0
	2	1	1
	3	0	1
	3a	0	1
	3c	1	0

*1, hemorrhagic transformation of infarcted brain tissue; 1a, HI1 scattered small petechiae, no mass effect; 1b, HI2 confluent petechiae, no mass effect; 1c, PH1 hematoma within infarcted tissue, occupying <30%, no substantive mass effect; 2, intracerebral hemorrhage within and beyond infarcted brain tissue; PH2, hematoma occupying 30% or more of the infarcted tissue, with obvious mass effect; 3, intracerebral hemorrhage outside the infarcted brain tissue or intracranial-extracerebral hemorrhage; 3a, parenchymal hematoma remote from infarcted brain tissue; 3b, intraventricular hemorrhage; 3c, subarachnoid hemorrhage; 3d, subdural hemorrhage; HI, hemorrhagic infarction; PH, parenchymatous hematoma. Only categories found in the study cohort are listed*.

As an additional analysis, we compared patients treated with EVT either with or without IVT with those 44 patients treated with IVT only (see Supplemental Table). In this comparison, IVT patients again had a higher premorbid Rankin scale, and premorbid disability was more frequent and so was diabetes. EVT patients again were treated later after onset but received thrombolysis earlier than IVT patients. Other baseline parameters did not differ significantly. Regarding ENI or any other outcome parameter, there was no significant difference between these groups.

In univariate analysis, patients with ENI had more often EVT and had higher initial NIHSS. Other parameters did not differ. The above-mentioned two parameters were included as covariates in a binary logistic regression for ENI where both initial NIHSS (OR, 1.082; CI, 1.027–1.141; *p* = 0.003) and EVT (OR, 2.726; CI, 1.055–7.044; *p* = 0.038) predicted ENI independently (see Table 4). As a sensitivity analysis, we also performed a multivariate model including other factors with presumed association with ENI, like age, proximal occlusion location, IVT, and time to treatment, but again only EVT (OR, 5.0; CI, 1.2–20.6; *p* = 0.03) and baseline NIHSS (OR, 1.1; CI, 1.0–1.2; *p* = 0.02) remained predictive factors for ENI.

**Table 4:** Univariate and bivariate analysis for the primary endpoint ENI (delta NIHSS ≥ 4 or post-NIHSS = 0.

**Parameter**	**Univariate analysis**			**Bivariate analysis**		
	**OR**	**Lower CI**	**Upper CI**	**OR**	**Lower CI**	**Upper CI**
EVT	2.83	1.12	7.13	2.76	1.055	7.04
NIHSS on admission	1.085	1.02	1.144	1.082	1.027	1.141

*EVT, endovascular therapy; OR, odds ratio; CI, 95% confidence interval*.

## Discussion

In this retrospective multicenter study comparing BMM and EVT with sole BMM in acute isolated unilateral occlusion of the posterior cerebral artery, EVT was safe and effective in terms of reperfusion and bleeding complications. Furthermore, an immediate clinical improvement could be observed more frequently after EVT, while long-term clinical outcome did not differ between groups. Both EVT and a higher NIHSS at baseline predicted ENI.

Occlusions of the posterior cerebral artery were excluded from recent randomized clinical trials on endovascular therapy to treat large vessel occlusion in acute ischemic stroke. Nevertheless, MT is increasingly performed in PCA occlusion (PCAO) as demonstrated by a growing number of retrospective studies (15–17). They consistently report high recanalization rates between 80 and 100% with low procedural complication rates between 4 and 7% and good clinical outcome at 90 days in 59–66%. In the present study, complete or near complete recanalization could be achieved in 54% of patients treated with EVT; 16% of these exhibited a residual stenosis, which was the underlying cause for incomplete recanalization in four of five patients, one of which had an Asian ethnicity while the others were Western European. Interestingly, none of the studies reporting MT in PCAO found local PCA stenosis as underlying cause, although the prevalence in AIS patients is reported with 10–16% (9). The relatively high prevalence of stenosis in the present study might be attributable to the fact that we did not exclude patients on the basis of their age. Furthermore, vessel changes due to EVT, i.e., dissection or severe vasospasm cannot be ruled out completely.

There was a non-significant trend for a higher frequency of any intracranial bleeding in the EVT group, while there was only one fatal parenchymal hemorrhage that occurred in the BMM group after thrombolysis. There were no angiographic signs of vessel perforation. There was one (4%) subarachnoid hemorrhage in the EVT group as a complication attributable to endovascular treatment. This is in keeping with previous studies of which two report no procedural complications (15, 17), Meyer et al. reported two dissections in 43 cases (13), and Lee et al. reported 1 in 15 cases where recanalization failed due to local complications (16).

In the present study, there was no difference between these groups with regard to good clinical outcome defined as a score between 0 and 2 on the modified Rankin scale after 90 days. There is only one other study so far comparing clinical outcome between these groups in patients with acute occlusions of the proximal PCA, i.e., in the P1 and P2 segment. The patient cohort in this retrospective study by Strambo et al. (14) is similar to the present one in terms of patient numbers and demographic parameters. The initial symptom severity was equivalent, and there was a similar proportion of patients with premorbid disability, i.e., a mRS >2. The authors did not find a significant difference in clinical outcome after 90 days between treatment groups, which is consistent with the present results. Moreover, they only report a trend for an increase in ENI defined as a drop of four points or more on the NIHSS after EVT compared to BMM, while this difference was significant in the present study. In addition to ENI and mRS at 90 days, Strambo et al. also report results of a differentiated neuropsychological assessment with evaluation of visual deficits, apraxia, neglect, memory, executive functions, and attention. Especially in PCAO, this is a sophisticated approach since these features are proprietary to the PCA territory and underrecognized by the NIHSS and even more by the mRS at the same time. Importantly, Strambo et al. observed better visual outcomes after EVT only (14). Unfortunately, the local databases that were accessed for our analysis were lacking these specific neurological outcome features, and hence, we were not able to reproduce the above finding. Moreover, we could not determine whether PCA strokes led to loss of the driving license, which has an enormous effect on activities of daily living but again is not reflected in outcome scores.

Most recently, a retrospective multicenter study reported findings comparing EVT and BMM with or without thrombolysis in patients with peripheral PCAO, i.e., P2–P4 segments, hence after exclusion of P1 occlusions (13). Consistent with the more peripheral location, the median NIHSS of five was even lower than in the present study and the aforementioned by Strambo et al. (14). Of note, in this largest PCAO-EVT cohort published so far, procedural complications were similarly low despite the more peripheral occlusion locations. Again, the proportion of patients with good clinical outcome did not differ between groups after 90 days. ENI was more frequent in EVT patients but only in the subgroup presenting with an NIHSS >10. This is in accordance with the results from the present multivariate regression analysis where the use of EVT predicted ENI, but the effect on ENI was also dependent on the level of the baseline NIHSS. This means that ENI is more likely to be present in patients with higher baseline NIHSS. The fact that this does not translate into good clinical outcome after 90 days likely indicates that the improvement is in a range that is only detected by the NIHSS but not the mRS as to be expected for deficits of cortical functions of the PCA territory.

### Limitations

This study has several limitations, with its retrospective uncontrolled and non-randomized design being the most prominent one. We can also not exclude a bias by indication, since treatment allocation in the participating centers was based on local SOPs and individual decisions of the treating physicians. Hence, treatment groups were not balanced, but the restricted number of patients did not allow for matching or propensity-score-based analysis. Furthermore, we stress in the present study and in that from Strambo et al. that there was a considerable proportion of patients with premorbid disability. We found a significant correlation between the premorbid mRS and the initial NIHSS, which might imply that the initial NIHSS in PCAO might also be, at least in part, attributable to premorbid disability.

## Conclusions

In conclusion, our study adds to the current evidence that EVT in isolated PCAO seems to be safe with regard to procedure-related complications and that EVT is associated with ENI, but ENI is influenced or dependent on the baseline NIHSS score. Furthermore, EVT does not seem to be associated with higher rates of long-term good outcome compared to the best medical management group. However, other features than the NIHSS and mRS, such as detailed visual and neuropsychological functions, for example, may need to be measured and evaluated as necessary in future studies to more reliably assess patient's benefits after PCAO.

## Data Availability Statement

The anonymized raw data supporting the conclusions of this article will be made available by the authors, without undue reservation.

## Ethics Statement

The studies involving human participants were reviewed and approved by Heidelberg University Ethics Committee. Written informed consent for participation was not required for this study in accordance with the national legislation and the institutional requirements.

## Author Contributions

CH and SN performed statistical analyses and drafted the manuscript. All authors retrieved and reviewed local data, reviewed and approved the final version of the manuscript.

## Conflict of Interest

CH is a consultant for Brainomix. TN received research support from Medtronic, SVIN. DK received a grant from Else Kröner-Fresenius Center for Digital Health. MM is a consultant for Medtronic, MicroVention, Stryker Neurovascular, phenox (money paid to the institution); has grants/grants pending from Balt, MicroVention (money paid to the institution); and received payment for lectures including service on speakers bureaus from Medtronic, MicroVention, Stryker Neurovascular. PR is a consultant for Boehringer and received lecture fees from Bayer, Boehringer Ingelheim, BMS, Daichii Sankyo, and Pfizer. SN is a consultant for Brainomix, Boehringer Ingelheim and received payment for lectures including service on speakers bureaus from Pfizer, Medtronic, and Bayer AG. The remaining authors declare that the research was conducted in the absence of any commercial or financial relationships that could be construed as a potential conflict of interest.

## Publisher's Note

All claims expressed in this article are solely those of the authors and do not necessarily represent those of their affiliated organizations, or those of the publisher, the editors and the reviewers. Any product that may be evaluated in this article, or claim that may be made by its manufacturer, is not guaranteed or endorsed by the publisher.

## References

[1] Zeumer H Hacke W Kolmann HL Poeck K. Local fibrinolysis in basilar artery thrombosis (author's transl). Dtsch Med Wochenschr. (1982) 107:728–31. 10.1055/s-2008-1070010 7075491

[2] Liu X Dai Q Ye R Zi W Liu Y Wang H. Endovascular treatment versus standard medical treatment for vertebrobasilar artery occlusion (BEST): an open-label, randomised controlled trial. Lancet Neurol. (2020) 19:115–22. 31831388 10.1016/S1474-4422(19)30395-3

[3] ESO-WSO 2020. Joint meeting abstracts. Int J Stroke. (2020) 15:3–752. 10.1177/1747493020963387

[4] Kleine JF Beller E Zimmer C Kaesmacher J. Lenticulostriate infarctions after successful mechanical thrombectomy in middle cerebral artery occlusion. J Neurointerv Surg. (2017) 9:234–9. 10.1136/neurintsurg-2015-012243 26940316

[5] Horie N Morofuji Y Iki Y Sadakata E Kanamoto T Tateishi Y. Impact of basal ganglia damage after successful endovascular recanalization for acute ischemic stroke involving lenticulostriate arteries. J Neurosurg. (2019) 132:1880–8. 10.3171/2019.3.JNS182909 31151109

[6] Grange S Grange R Garnier P Varvat J Marinescu D Barral F-G. Boundary and vulnerability estimation of the internal borderzone using ischemic stroke lesion mapping. Sci Rep. (2020) 10:1662. 10.1038/s41598-020-58480-y 32015357 PMC6997399

[7] Milandre L Brosset C Botti G Khalil R. A study of 82 cerebral infarctions in the area of posterior cerebral arteries. Rev Neurol. (1994) 150:133–41. 7863153

[8] Johansson T. Occipital infarctions associated with hemiparesis. Eur Neurol. (1985) 24:276–80. 10.1159/000115807 4006996

[9] Brandt T Steinke W Thie A Pessin MS Caplan LR. Posterior cerebral artery territory infarcts: clinical features, infarct topography, causes and outcome. Multicenter results and a review of the literature. Cerebrovasc Dis. (2000) 10:170–82. 10.1159/000016053 10773642

[10] von Kummer R Broderick JP Campbell BC Demchuk A Goyal M Hill MD. The Heidelberg bleeding classification: classification of bleeding events after ischemic stroke and reperfusion therapy. Stroke. (2015) 46:2981–6. 10.1161/STROKEAHA.115.010049 26330447

[11] Zaidat OO Yoo AJ Khatri P Tomsick TA von Kummer R Saver JL. Recommendations on angiographic revascularization grading standards for acute ischemic stroke: a consensus statement. Stroke. (2013) 44:2650–63. 10.1161/STROKEAHA.113.001972 23920012 PMC4160883

[12] Amiri H Bluhmki E Bendszus M Eschenfelder CC Donnan GA Leys D. European cooperative acute stroke study-4: extending the time for thrombolysis in emergency neurological deficits ECASS-4: ExTEND. Int J Stroke. (2016) 11:260–7. 10.1177/1747493015620805 26783318

[13] Meyer L Stracke CP Jungi N Wallocha M Broocks G Sporns PB. Thrombectomy for primary distal posterior cerebral artery occlusion stroke: the TOPMOST study. JAMA Neurol. (2021) 78:434–44. 10.1001/jamaneurol.2021.0001 33616642 PMC7900924

[14] Strambo D Bartolini B Beaud V Marto JP Sirimarco G Dunet V. Thrombectomy and thrombolysis of isolated posterior cerebral artery occlusion: cognitive, visual, and disability outcomes. Stroke. (2020) 51:254–61. 10.1161/STROKEAHA.119.026907 31718503

[15] Memon MZ Kushnirsky M Brunet MC Saini V Koch S Yavagal DR. Mechanical thrombectomy in isolated large vessel posterior cerebral artery occlusions. Neuroradiology. (2021) 63:111–6. 10.1007/s00234-020-02505-w 32748080

[16] Lee HN Kim BT Im SB Hwang SC Jeong JH Chung MY. Implications of mechanical endovascular thrombectomy for acute basilar and posterior cerebral artery occlusion. J Cerebrovasc Endovasc Neurosurg. (2018) 20:168–75. 10.7461/jcen.2018.20.3.168 30397588 PMC6199399

[17] Clarencon F Baronnet F Shotar E Degos V Rolla-Bigliani C Bartolini B. Should posterior cerebral artery occlusions be recanalized? Insights from the Trevo Registry. Eur J Neurol. (2020) 27:787–92. 10.1111/ene.14154 31997505

