# Supplementary material for: Mechanical Thrombectomy in Isolated Occlusion of the Proximal Posterior Cerebral Artery
Source: Front Neurol. 2021 Jul 29;12:697348. doi: 10.3389/fneur.2021.697348 (PMC8358070; doi:10.3389/fneur.2021.697348)
Supplement: Supplementary file 1 [file Table_1.DOCX]

| **baseline clinical data** | **EVT ± IVT (23)** | **IVT (44)** | **p-value** |
| --- | --- | --- | --- |
| sex (female) | 9 (39.1%) | 24 (54.5%) | 0.30^X^ |
| age, mean +/-SD | 70 ± 13.3 | 69 ± 16.7 | 0.43^#^ |
| mothership (vs “drip & ship”) | 21 (91.3%) | 38 (86.4%) | 0.70^X^ |
| wakeUp Stroke | 7 (30.7%) | 6 (13.6%) | 0.11^X^ |
| premorbid mRS, median (range) | 0 (0-4) | 1 (0-4) | 0.006 |
| premorbid disability (mRS>2) | 1 (4.3%) | 11 (25%) | 0.046^#^ |
| NIHSS on admission, median (range) | 9 (1-20) | 7 (1-32) | 0.61^#^ |
| hypertension | 16 (69.6%) | 39 (88.6%) | 0.09^X^ |
| diabetes | 4 (17.4%) | 22 (50%) | 0.02^X^ |
| hypercholesterolemia | 8 (36.4%) | 23 (52.3%) | 0.30^X^ |
| current smoking | 5 (22%) | 7 (15%) | 0.74^X^ |
| previous stroke | 6 (27.3%) | 11 (25%) | 1.0^X^ |
| coronary heart disease | 8 (34.8%) | 13 (29.5%) | 0.78^X^ |
| atrial fibrillation | 6 (26.1%) | 20 (45.5%) | 0.19^X^ |
| oral anticoagulation | 4 (17.5%) | 3 (6.8%) | 0.22^X^ |
| mean arterial pressure | 110+/-22 | 112 ± 15.9 | 0.87^§^ |
| **baseline radiological findings** | | | |
| occlusion location, proximal | 16 (69.9%) | 29 (65.9%) | 1.0^X^ |
| CT performed | 18 (78.3%) | 38 (86.4%) | 0.49^X^ |
| MRI performed | 5 (21.7%) | 12 (27.3%) | 0.77^X^ |
| perfusion imaging performed (CTP or MRP) | 10 (43.5%) | 21 (47.7%) | 0.80^X^ |
| MRP/CTP mismatch >20% | 9/10 (90%) | 14/21 (70%) | 0.37^X^ |
| **procedural parameters** |  |  |  |
| intravenous thrombolysis | 5 (21.7%) | 44 (100%) | <0.001^X^ |
| Time-to-treatment (time from symptoms onset to IVT or to Groin Puncture) in min, mean+/-SD | 353 ± 263 | 176 ± 115 | 0.016^§^ |
| Onset to Door (EVT center) in min, mean+/-SD | 281 ± 223 | 132 ± 94 | 0.07^§^ |
| Door To Needle time in min, mean+/-SD | 20 ± 13 | 61 ± 37 | 0.018^§^ |
| Door To Groin time in min, mean+/-SD | 122 ± 100 | n.a. | n.a. |
| **outcome parameters** | | | |
| Recanalization complete or near-complete (TICI 2b-3) | 58% (14/24) | n.a. | n.a. |
| Residual artery stenosis | 16% (4/25) | n.a. | n.a. |
| NIHSS drop, median (IQR) | 4 (-1 - 7) | 3 (0-7) | 0.66^#^ |
| ENI | 14 (61%) | 19 (43.2%) | 1.0^X^ |
| mRS at day 90, median (range) | 3 (0-6) | 3 (0-6) | 0.74^#^ |
| good outcome incl. return to baseline mRS | 10 (43.5%) | 14 (31.8%) | 0.42^X^ |
| Excellent outcome (mRS 0-1 at 90 days) | 4 (17.4%) | 6 (13.6%) | 0.73^X^ |
| symptomatic ICH | 6 (26.1%) | 6 (13.6%) | 0.31^X^ |
| Fatal ICH | 0% | 1 (2.3%) | 1.0^X^ |
| in-house deaths | 1 (4.3%) | 3 (6.8%) | 0.57^X^ |

Supplemental table - Baseline characteristics & procedural and outcome parameters (IVT=intravenous therapy; EVT=endovascular therapy; ENI=early neurological improvement; ICH=intracranial hemorrhage; χ=chi-square-test, MWU= Mann Whitney Test)
